# Supplementary material for: Accurate Early Detection and EGFR Mutation Status Prediction of Lung Cancer Using Plasma cfDNA Coverage Patterns: A Proof-of-Concept Study
Source: Biomolecules. 2024 Jun 17;14(6):716. doi: 10.3390/biom14060716 (PMC11202186; doi:10.3390/biom14060716)
Supplement: Supplementary file 1 [file biomolecules-14-00716-s001.zip › Supplementary.pdf]

Supplementary

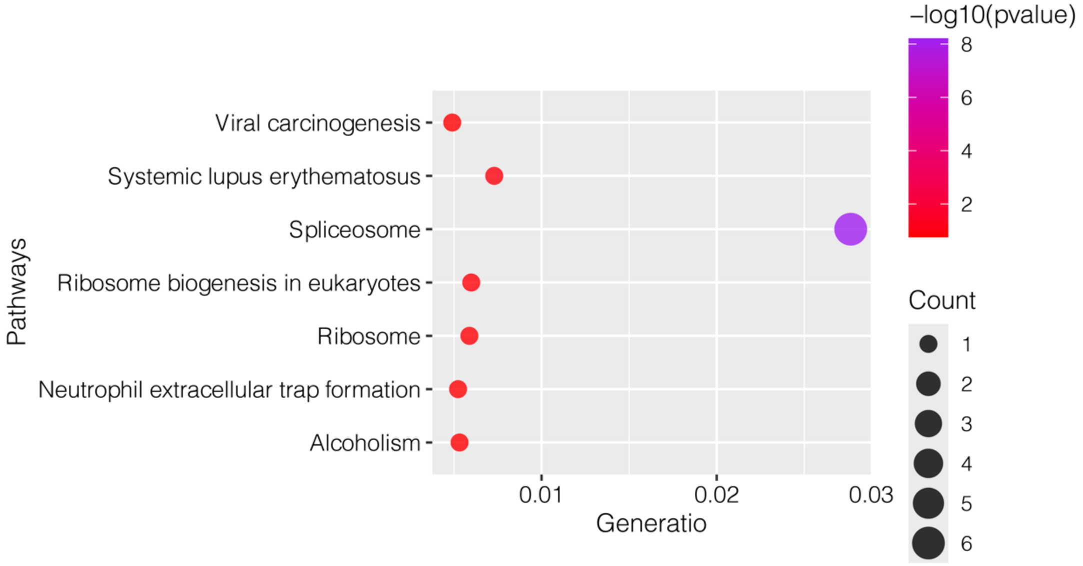

Fig S1 | Gene enrichment analysis result of differentially expressed genes between stages I/II and stages III/IV in TCGA RNA dataset.

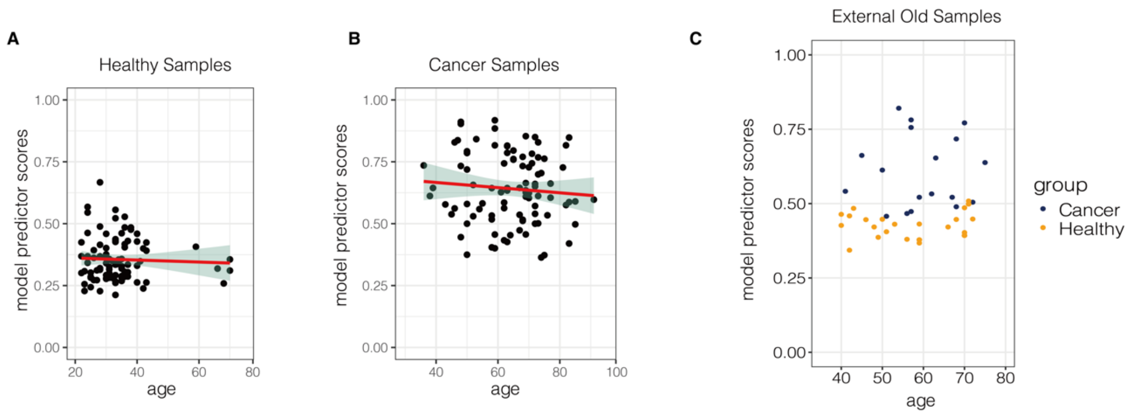

Fig S2 | **The relationship between age and predictor scores. A-B.** It illustrated the relationship between age and predictor scores within the healthy and cancer samples of the model building cohort, respectively. Additionally, each part includes a fitted line with confidence intervals to enhance clarity. **C.** It illustrated the model's good

prediction performances on a selection of balanced, aged external samples from both cancer and healthy groups.

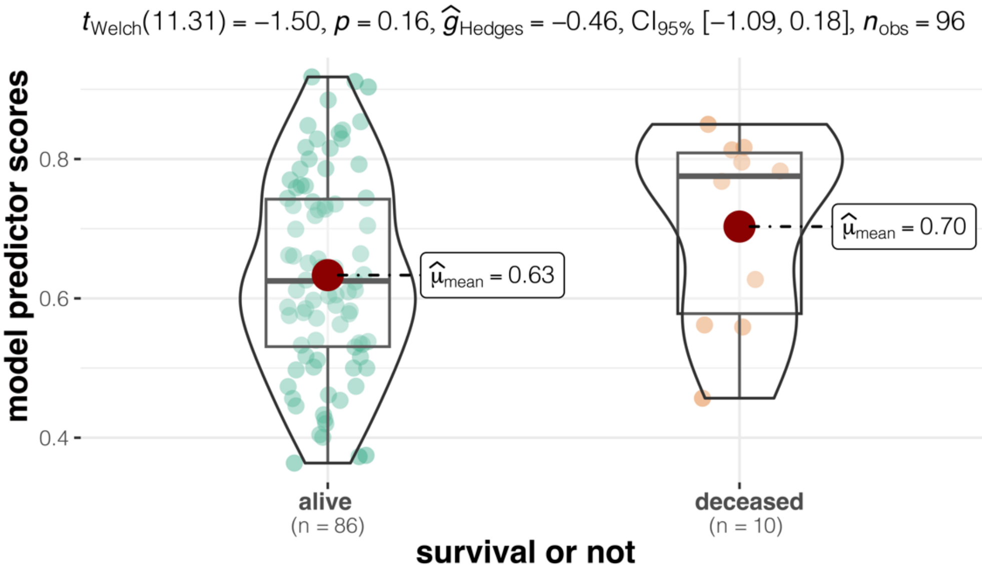

Fig S3 | Early Cancer Screening Model's predictor scores between deceased and alive cases.
